# Reverse-Bent Modular Coil Structure with Enhanced Output Stability in DWPT for Arbitrary Linear Transport Systems

**DOI:** 10.3390/s24227171

**Published:** 2024-11-08

**Authors:** Jia Li, Chong Zhu, Junyi Ji, Jianquan Ma, Xi Zhang

**Affiliations:** 1 School of Mechanical and Engineering, Shanghai Jiao Tong University, Shanghai 200240, China; 18852647159@sjtu.edu.cn (J.L.); jijunyi@sjtu.edu.cn (J.J.); braver1980@sjtu.edu.cn (X.Z.); 2 School of Traffic and Transportation, Bingtuan Xingxin Vocational and Technical College, Xinjiang 841007, China; majq@btc.edu.cn

**Keywords:** dynamic wireless power transfer, coil structure, linear transport systems

## Abstract

Dynamic wireless power transfer (DWPT) systems with segmented transmitters suffer from output pulsations during the moving process. Although numerous coil structures have been developed to mitigate this fluctuation, the parameter design process is complicated and restricted by specific working conditions (e.g., air gaps). To solve these problems, a novel reverse-bent modular transmitter structure is proposed for DWPT in industrial automatic application scenarios such as linear transport systems. Considering the heterogeneous current density distribution in the adjacent region between two coils which causes a drop in magnetic field, the proposed coil structure attempts to eliminate the effects of the adjacent region by bending the terminal parts of each coil reversely to the ferrite layer for shielding. Compared to traditional planar couplers, this structure array can generate a uniform magnetic field over various air gaps. A 100 W laboratory prototype was built to verify the feasibility of the proposed system. The experimental results show that the proposed system achieved a constant output voltage, and the output pulsation was within ±2.3% in the dynamic powering process. The average efficiency was about 88.29%, with a 200 mm transfer distance. When the air gap varied from 20 mm to 30 mm, the system could still retain constant voltage output characteristics.

## 1. Introduction

Dynamic wireless power transfer (DWPT) technology has been widely applied in various scenarios. Compared to traditional stationary wireless power transfer technology applied for consumer electronics, electric vehicles, and medical implants [1,2,3,4,5], dynamic wireless power transfer systems can provide real-time power to moving loads such as roadway vehicle charging [6] and automated material handling devices in factories [7,8].

**Figure 1:**
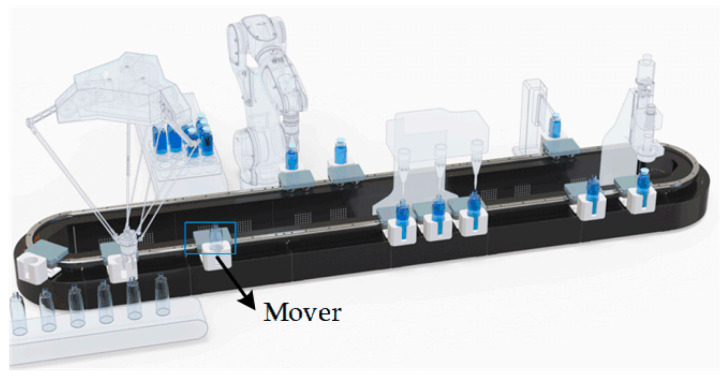
Schematic diagram of magnetically levitated transport systems [12].

Generally, DWPT systems are suitable for applications such as electric vehicles and automatic guided vehicles for charging, which have been extensively studied. Further, they are also a promising technology for improving mobility in emerging mechatronic applications [9] such as magnetically levitated transport systems. As a replacement of conventional conveyors, magnetically driven transport systems are deployed to transport items to machines to carry out production tasks [10,11]. For magnetically driven transport systems, every mover is driven by an electromagnetic force and can be controlled independently to streamline the production flow without heavy batteries or cables. Furthermore, the mover can also be extended to serve as a mobile processing station with sensors powered by WPT technology with the benefit of no recharging, higher utilization efficiency, and a lower cost. Movers with DWPT make it possible to process and check the quality of products on the mover while the process is running, as shown in Figure 1. Nevertheless, for such DWPT systems, a low output voltage fluctuation must be guaranteed. In addition, the modular design should also be taken into consideration to adapt to various transportation line requirements by flexible assembly.

Several studies have been carried out to design magnetic couplers for DWPT systems. Based on the structure of the transmitter, the DWPT system can be divided into two categories. One is to make use of a single long Tx coil for power transmission [13,14,15,16]. However, during the working process, such a system exhibits high coupling leakage and thus suffers from significant coil loss and poor efficiency [7]. Furthermore, the magnetic field emission from Tx coils could incur higher system sensitivity to nearby metallic objects, causing more interference with other electronic devices.

Accordingly, the short-individual transmitter type has been proposed for DWPT systems. Compared to the long-track type, the whole Tx coils are composed of arrays of short Tx coils which are the same size as that in the stationary WPT system [17,18,19,20,21,22,23,24,25,26,27]. Multiple short Tx coils are arranged in an array along the conveyor track. Since the transmitters can be excited or turned off according to the receiver position, the short-individual transmitter type has the advantage of high coil efficiency and a lower magnetic field emission. However, such a segmented arrangement inevitably causes voltage pulsations while the receiver is moving. The mutual inductance drops in the region between the transmitters, and the output voltage fluctuates when the receiver moves to the segmented region. For example, in [17], the circular coil pad is applied for power transfer. The output power reduces to nearly zero in the segmented region.

For battery-less DWPT applications, the output voltage pulse due to the change in the mutual inductance harms the electronic devices (overvoltage or undervoltage). Therefore, the output voltage must be stable. To smooth the variation in the output voltage, a variety of methods have been proposed. The first is to optimize the structure of the receivers. In [19], a three-phase DWPT system is proposed to gain a constant voltage with a fluctuation of about 1.17%. The length of the receiver coil is three times longer than the transmitter coil to maintain a stable output voltage. In [20], a two-phase rectifier with two Rx coils is adopted, but the Rx coils must be placed away from each other to avoid coupling. Further, in [21], a triple-coil receiver with a triple-diode rectifier structure is proposed with a fluctuation of only about 3.49%. The above analysis indicates that receivers are usually enlarged for output stability. However, the size of the receiver is restricted by the moving bases. For magnetically levitated movers, a small size is usually required to satisfy flexible motion profiles.

Secondly, transmitters can also be optimized. In [22,23], the transmitters are placed closely to strengthen the magnetic field between two transmitters. However, the power pulsation still exists. Further, in [24], the rectangular transmitters are arranged one by one, in which the self-couplings between Tx coils should be taken into consideration. The output power pulsation can be within ±2.9% of the average power by properly designing the coil structure. The literature in [25] proposes a new coupler with unipolar and bipolar coils laid alternatively to eliminate cross-couplings. The output fluctuations are within ±2%. However, the optimization of the transmitters requires a time-consuming simulation process by Maxwell. And, the size of the receiver is severely restricted by the transmitters. In [26], magnetic integration is implemented to smooth the coupling variation. The additional coupling coils make the design process more complicated. In [27], an enhanced I-shaped transmitter is adopted. The output voltage variation is only ±1.03%, with an efficiency ranging from 87.22% to 87.98%. However, the design process is based on a set of specific parameters, such as the air gap and size of the coils. In other words, most of these design structures just focus on one specific working condition. If the air gap or the receiver size changes, the coil parameters need to be redesigned.

Existing research on DWPT output power pulsation for magnetically levitated movers is insufficient. On the one hand, FEM analysis is often required to search for a specific optimal solution for the coil structure, which takes a large amount of computational time. And, the designed DWPT system can only be applied for a specific working condition, such as a fixed transfer distance and fixed receiver size. On the other hand, the size of the receiver should be limited for magnetically levitated mover applications. Thus, one of the main challenges is to design a transmitter coil structure with a smooth magnetic field in all directions naturally. Meanwhile, the design procedure should be simple and efficient. Consequently, based on the commonly used rectangular coils, a novel short-individual transmitter coil structure is proposed in this article, providing a low-cost and low-fluctuation solution for the DWPT system.

In this article, a coil structure arrangement mode with terminal parts reversely bent is proposed for a short-individual transmitter type DWPT system. With the magnetic field confinement of the ferrite layer, the bent parts have few effects on power transfer region. Then, by placing the coils closely, the transmitter array can be considered a segmented long-track-type transmitter. Compared to traditional planar coils, the proposed transmitter structure can generate a uniform magnetic field at different transmission gaps, which means that the system adapts to various working conditions and receiver sizes rather than a specific operating scenario with fixed air gaps. Compared to previous research, the proposed coil structure design procedure is very simple, without any iterative optimization by simulation. And, the magnetic field flatness along the track can be guaranteed at various gaps. The coil arrangement is suitable for linear transport systems such as magnetically levitated moving bases, in which the receiver size is limited. At the same time, magnetic field suppression methods are discussed. With the designed coil structure, the output voltage pulsation is within ±2.3% of the average voltage.

## 2. Proposed Coil Structure for DWPT

This article adopts short-individual transmitters for DWPT. In this section, the structure and magnetic flux distribution of the proposed transmitters are provided. Finite-element analysis (FEA) simulations are performed with Maxwell for verification.

### 2.1. Structure Design

**Figure 2:**
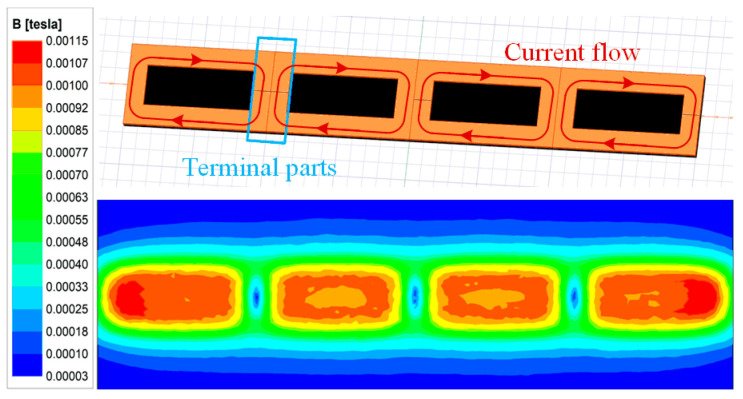
Magnetic field for traditional planar coils.

**Figure 3:**
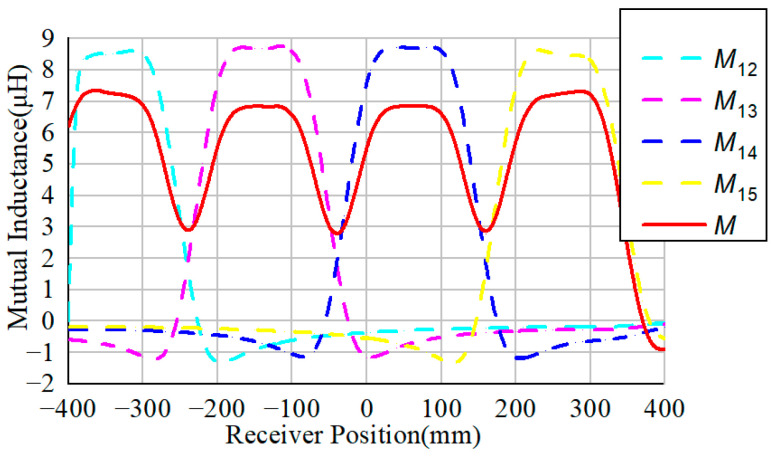
Mutual inductance for traditional planar coils.

Figure 2 and Figure 3 show the Z-direction magnetic field distribution and mutual inductance of traditional planar coils. Obviously, the magnetic flux density maintains almost constant among each coil but decreases sharply along the terminal parts. Since the terminal parts share two currents flowing in opposite directions, they will decrease the magnetic flux density of the corresponding region framed by the red box.

**Figure 4:**
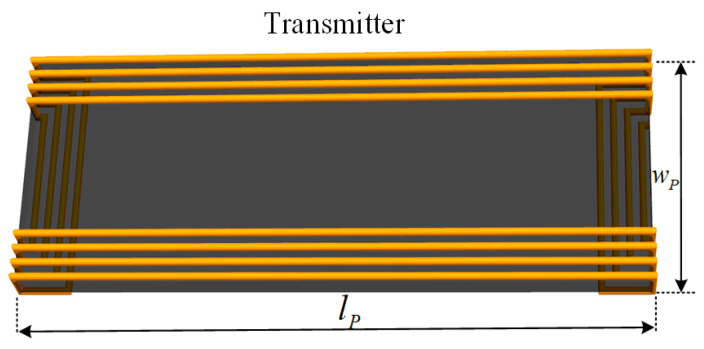
Proposed coil structure.

**Figure 5:**
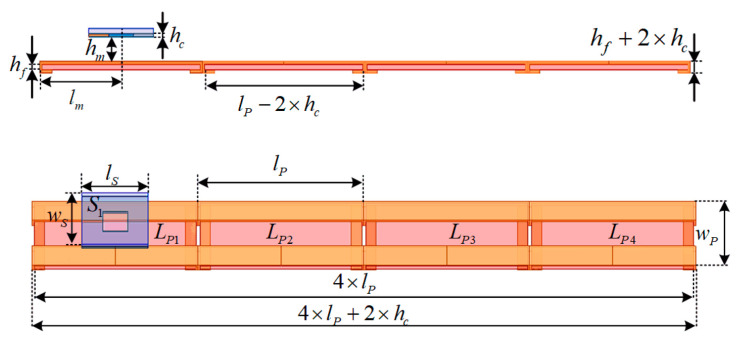
Layout of the proposed magnetic couplers.

To deal with the problem, this article proposes a new coil structure, as shown in Figure 4. Compared to traditional segmented rectangular Tx coils, the proposed transmitters can be regarded as a unipolar rectangular structure with two terminal sides bent to the back of ferrite plates, and the magnetic field generated by the bent parts of the transmitter is concentrated in the ferrite region. The layout of the whole magnetic couplers is shown in Figure 5. The parameters are defined in Table 1. The receiver coil is a unipolar structure, labeled *S*_1_. Four unipolar transmitters labeled *L_P_*_1_ to *L_P_*_4_ are arranged closely to couple with the receiver. Thus, the segmented transmitters can perform as long-track-type transmitters with stable coupling characteristics.

**Figure 6:**
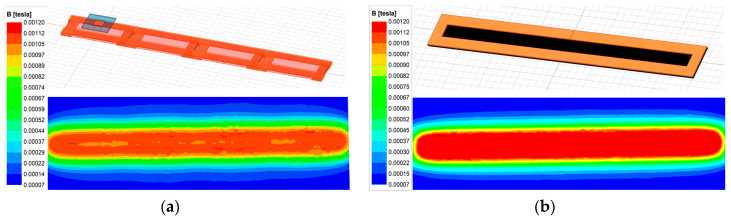
Magnetic field simulated by Maxwell. (**a**) For proposed transmitters when *h*_m_ = 20 mm. (**b**) For long-track-type transmitter when *h*_m_ = 20 mm.

Further, the magnetic field distribution of the transmitters is simulated as an example, as shown in Figure 6a. The magnetic field of the corresponding long-track-type transmitter with the same size is also simulated in Figure 6b. Obviously, the magnetic field distribution between the two transmitters is similar. Therefore, the proposed segmented transmitters can be considered a long-track-type transmitter for power transfer while avoiding extra magnetic flux emissions by controlling the transmitters to be turned on or off.

### 2.2. Transmitter Design

**Figure 7:**
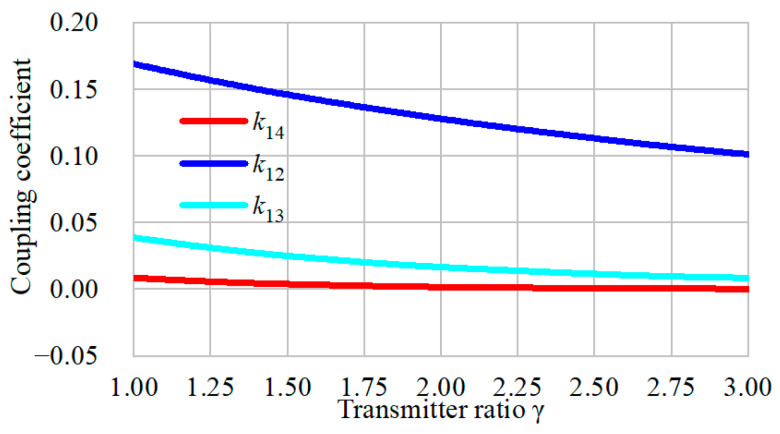
Coupling coefficient when the transmitter length varies.

Here, Litz wire with diameter of 2.5 mm is used to build the coil. Ferrite plates are also applied with the transmitters and receivers to strengthen the magnetic coupling while avoiding magnetic saturation. Since the size of the receiver is restricted for application, it can be wrapped with two layers to increase the self-inductance. Figure 5 also shows that the length of the transmitter is larger than the width. By extending the length, only one or two transmitters are switched to couple with the receiver, and the total cost of the Litz wire is lower. However, the overall efficiency may decrease. Due to the space limitation, the transmitter width *w*_p_ is set to 100 mm, and each coil has twelve turns. The length of the transmitter can be varied. γ is defined as *l*_p_/*w*_p_. Obviously, when the ratio is set to be large, the overall efficiency may decrease. Here, the ratio is set to be more than 3. The FEA-simulated results of the coupling coefficient between the transmitters are shown in Figure 7. The four transmitters are excited by currents of the same direction and amplitude.

**Figure 8:**
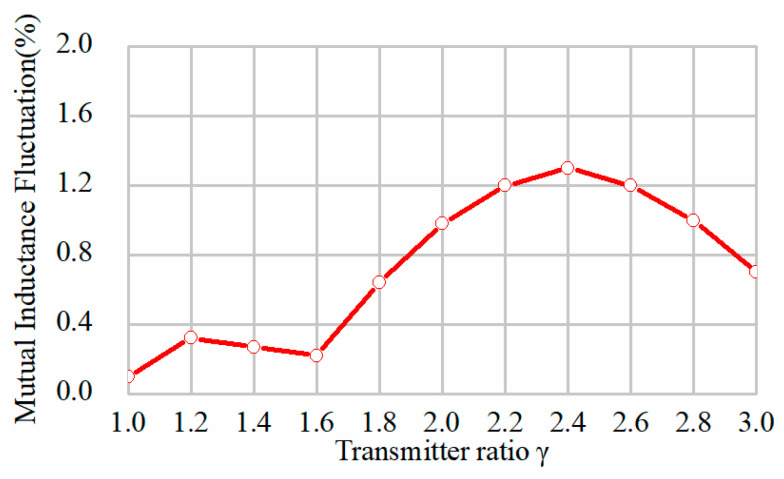
Curves of mutual inductance fluctuation with transmitter length by simulation.

As shown in Figure 7, the coupling coefficient between the adjacent transmitters is *k*_12_, and the other symbols indicate the coupling coefficients with nonadjacent coils. All the transmitters are placed closely without overlap or interspace. Therefore, the signs of the coupling coefficients are all positive. Figure 6 shows that the coupling coefficient decreases with the parameter *l*_p_. It indicates that, when the length of transmitter increases, the cross-coupling effect becomes weak. With a further increasing length, the cross-coupling is eliminated. The couplings between the nonadjacent transmitters are much smaller than the adjacent ones. When the transmitter ratio is 2, it turns out that $k_{13}=0.016$ and $k_{14}=0.002$, which means that the coupling with nonadjacent coils can be neglected. However, the transmitter cannot be fully decoupled from adjacent coils. Furthermore, in this system, since the bent parts transfer little power to the receiver, the total effective region of the transmitter for the power transfer may decrease, and if γ increases, the loss will be reduced. Another significant aspect to consider is the mutual inductance variation when the transmitter length varies. The simulated mutual inductance fluctuation of the proposed transmitters has been obtained, as indicated in Figure 8. It is observed that the fluctuations are quite low when the transmitter ratio ranges from 1 to 3. The overall fluctuation is within 1.5%, which is always acceptable in this application. Nonetheless, more materials are required to cover the same total length when the ratio decreases.

For practical applications, the overall efficiency of the transmitters should be taken into consideration. Therefore, the segmented coil length *l*_P_ cannot be too large. According to be above analysis, the transmitter ratio is set to 2. This placement could effectively make use of the coil length and keep a relatively high efficiency while decreasing the cross-coupling between the coils, and the mutual inductance fluctuation is around ±0.6%.

### 2.3. Dynamic Performance

According to the above-mentioned conclusions, the parameters of the proposed structure are shown in Table 2. In this article, the DWPT system is designed for magnetically levitated movers; thus, the planar square receiver/ferrite length *l*_s_ and width *w*_s_ are designed to be identical at 80 mm. The turns are set as 20, with two layers for increasing the self-inductance. As for the transmitters, the length *l*_p_ is 200 mm, and the width is 100 mm. The number of turns is 12. Both the receiver and transmitters are manufactured by Litz wire, with diameters of 2.5 mm. Considering the space limitation, the air gap between the transmitters and receiver ranges from 20 mm to 30 mm. The thickness of the ferrites for the transmitters and receiver are all set to 5 mm to avoid magnetic saturation.

**Figure 9:**
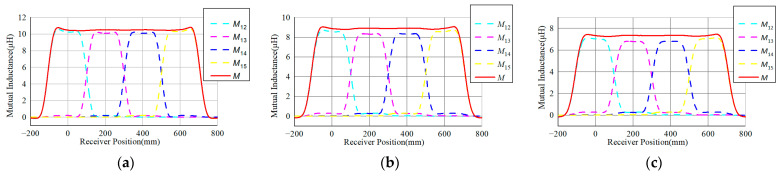
Simulated mutual inductances between the receiver and transmitters (**a**) when *h*_m_ = 20 mm; (**b**) when *h*_m_ = 25 mm; (**c**) when *h*_m_ = 30 mm.

Utilizing the Maxwell simulation, the corresponding mutual inductance of the receiver with different transmitters is depicted in Figure 9. When the receiver moves along the transmitters, each transmitter coil can transfer power through the magnetic coupling. The sum of the transferred power is the total power. To minimize the variation in the output power in the dynamic process, the total mutual inductance variation should be minimal.

When the air gap is 20 mm, the resulting mutual inductance between the transmitters and receiver is shown in Figure 9a. The dashed lines represent the variation in the mutual inductance with each transmitter, identified as *M*_1_, *M*_2_, *M*_3_, and *M*_4_. The total mutual inductance could then be defined as follows:

$$M=M_{1}+M_{2}+M_{3}+M_{4}$$

The total mutual inductance *M* is shown as a solid red line. It can be observed from Figure 9a that the mutual inductance variation is quite small in the middle section of the transmission array. The fluctuation rate is within ±0.25% in the middle section when *l*_m_ ∈ [200 mm, 400 mm], while in the interval when *l*_m_ ∈ [0 mm, 600 mm], it is within ±0.6%. Compared to the structure in the literature [26,27], the proposed DWPT structure has a lower variation along the transmission array. Furthermore, when the air gap is 25 mm and 30 mm, the corresponding results are shown in Figure 9b,c. The variation in the mutual inductance when *h*_m_ equals 25 mm is about ±0.26% in the middle section, while it is ±0.85% in the interval when *l*_m_ ∈ [0 mm, 600 mm]. When *h*_m_ increases to 30 mm, the fluctuation is within ±0.25% in the middle section, and the fluctuation increases to about ±0.9% in the interval when *l*_m_ ∈ [0 mm, 600 mm]. Obviously, the variation along the transmitter array is maintained at around ±0.25% in the middle section regardless of the air gap distance.

### 2.4. Magnetic Field Suppression Method

Normally, for DWPT systems, the metal objects around the magnetic field may be heated due to the eddy current effect. For the proposed transmitter structure applied in linear transport systems, the magnetic flux under the transmitter may heat the track. The temperature rise may cause damage to the electric elements or make the control systems under the transmitters break down. Therefore, suppressing the magnetic field under the transmitter can help the linear transport system operate safely and efficiently.

**Figure 10:**
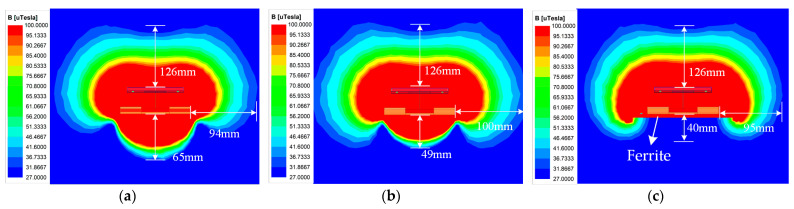
Magnetic field simulated by Maxwell when *l*_m_ = 400 mm for proposed transmitters. (**a**) With one layer in bent parts. (**b**) With two layers in bent parts. (**c**) With another ferrite layer.

The magnetic coupler design is a crucial method to achieve magnetic field suppression. Obviously, the reversely bent part of the coils contributes most of the magnetic flux under the transmitters. The magnetic field distribution when *l*_m_ equals 400 mm is depicted in Figure 10. As shown in Figure 10a, the magnetic induction intensity under the transmitter is relatively small compared to the power transfer region. To further achieve magnetic field suppression, the bent part is wrapped to be two layers with six turns per layer. The corresponding magnetic field distribution is shown in Figure 10b. Normally, the exposure limit of magnetic fields for the general public is 27 μT. It is observable that the distance decreases from 65 mm to 49 mm. Further, by adding another ferrite layer under transmitters, the magnetic field under the transmitters can be concentrated to ease the effects of the eddy current for linear transport systems, as shown in Figure 10c. Since the bent parts can be considered another layer of coils, the structure of the Tx coil can be composed of one coil layer, one ferrite layer, one coil layer. The top coil layer contributes magnetic flux to the power transfer region, while the bent coil layer may contribute magnetic flux to the track region. Therefore, another ferrite layer could be applied to form a coil–ferrite–coil–ferrite structure to ease the magnetic flux extended to track region.

## 3. *LCC-S* Compensation Circuit Design

Typically, *LCC* compensation topology is preferred in transmitters as it can perform as a current source to reduce the fluctuation in the transmitter [24]. In the proposed DWPT system, *LCC-S* topology is adopted to perform as a constant voltage source. Since the receiver is small, only two transmitters may work together to transfer power when the receiver moves.

**Figure 11:**
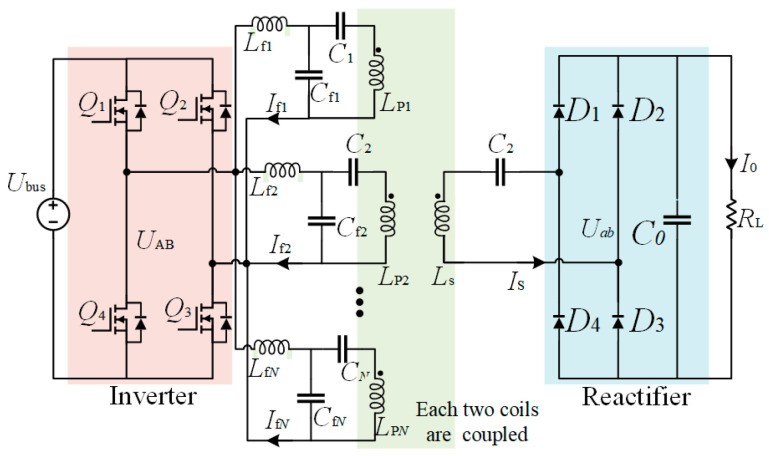
Topology of *LCC-S* DWPT system.

**Figure 12:**
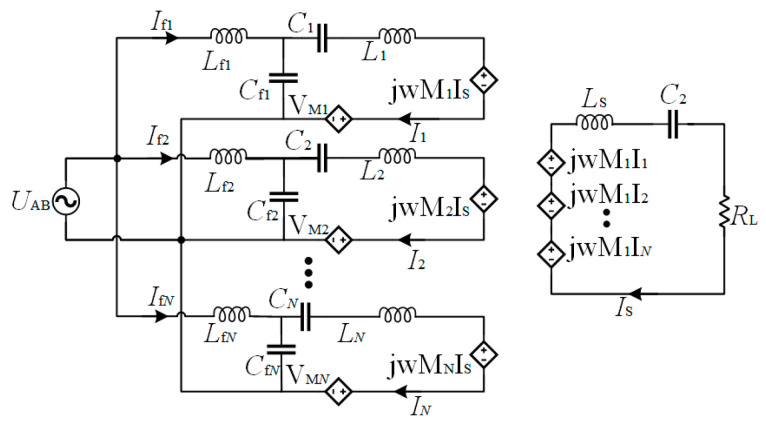
Simplified resonant circuit.

The configuration of the system is illustrated in Figure 11. The system is powered by the dc voltage source *V*_dc_, which is converted to the high-frequency ac voltage *V*_in_ by a full-bridge inverter. There are *N* sets of *LCC*-compensated transmitters with parallel connections on the primary side to transfer power. *L*_fi_, *C*_fi_, and *C*_i_ are the inductance and capacitance that constitute the primary *LCC* tank, and *L*_pi_ represents the self-inductance of the transmitter. *L*_s_ is the self-inductance of the receiver, which is compensated by the capacitor *C*_s_. *M*_psi_ refers to the mutual inductance between *L*_pi_ and *L*_s_, and *M*_ij_ is the cross-coupling between the transmitters. *R*_L_ is the load resistance. Since the *LCC* network behaves as a low-pass filter, the fundamental harmonious approximation (FHA) method is adopted to analyze the system. The simplified circuit is shown in Figure 12.

The parasitic resistances of the passive components are neglected for simplification. The parameters of the primary side are designed to be

(1) $$C_{fi}=1/\left(\omega^{2}L_{fi}\right),i=1,2\ldots ,N$$

According to the primary voltage loop, we can obtain

(2) $$V_{AB}=j\omega L_{fi}I_{fi}+\frac{1}{j\omega C_{fi}}(I_{fi}-I_{i}),i=1,2\ldots ,N$$

Given the parallel resonance of *C*_fi_ and *L*_fi_, considering (1) and (2), the current *I*_i_ can be deduced as

(3) $$I_{i}=\frac{V_{AB}}{j\omega L_{fi}},i=1,2\ldots ,N$$

In order to simplify the analysis, the compensated inductance and corresponding capacitors are designed to be the same, and the parameters satisfy

(4) $$\{\begin{array}{c} L_{f1}=L_{f2}=\ldots =L_{fN}=L_{f}\\ C_{f1}=C_{f2}=\ldots =C_{fN}=C_{f}\\ L_{1}=L_{2}=\ldots =L_{N}=L_{P} \end{array}$$

Considering (3) and (4), it is found that the transmitter current keeps the same for each transmitter, which can be expressed as *I*_P_.

Then, according to Kirchhoff’s voltage law, the system can be described as

(5) $$\{\begin{array}{c} V_{AB}=j\omega L_{fi}I_{fi}+(\frac{1}{j\omega C_{i}}+j\omega L_{i})I_{i}+j\omega M_{i}I_{s}+V_{Mi}\\ \sum_{i=1}^{N}j\omega M_{i}I_{i}=(j\omega L_{s}+\frac{1}{j\omega C_{s}}+R_{L})I_{S} \end{array},i=1,2\ldots ,N$$

The parameter relationship is expressed as

(6) $$C_{s}=1/(\omega^{2}L_{s})$$

On the primary side, since the transmitters couple with each other, the coupled voltage with the other transmitters can be expressed as

(7) $$V_{Mi}=\sum_{q=1,q\neq i}^{N}j\omega M_{iq}I_{P},i=1,2\ldots ,N$$

By substituting (3), (6), and (7) into (5), the current can then be solved as

(8) $$I_{fi}=\frac{V_{AB}}{j\omega L_{f}}(1-\frac{L_{P}}{L_{f}}+\frac{1}{\omega^{2}L_{f}C_{i}}-\sum_{q=1,q\neq i}^{N}\frac{M_{iq}}{L_{f}}+j\frac{\omega M_{i}}{R_{L}}\sum_{i=1}^{N}M_{i})$$

It can be found from (8) that the input impedance is influenced by the cross-couplings between the transmitters. Therefore, to eliminate the reactive part of the impedance, the value of capacitor *C*_i_ can satisfy

(9) $$1-\frac{L_{P}}{L_{f}}+\frac{1}{\omega^{2}L_{f}C_{i}}-\sum_{q=1,q\neq i}^{N}\frac{M_{iq}}{L_{f}}=0$$

By solving (9), *C*_i_ is given by

(10) $$C_{i}=\frac{1}{\omega^{2}(L_{P}-L_{f}+\sum_{q=1,q\neq i}^{N}M_{iq})},i=1,2\ldots ,N$$

By substituting (10) to (5), the output voltage is calculated as

(11) $$V_{ab}=I_{S}R_{L}=\frac{V_{AB}}{L_{f}}\sum_{i=1}^{N}M_{i}$$

From the above analysis, it is shown that the output voltage is composed of multiple *LCC-S* systems. The receiver only picks up energy from the transmitters that are coupled with the receiver. According to the dynamic performance in Section 2, at most two *LCC-S* systems are turned on for transferring power when the receiver is in the adjacent region in this article. By a parallel connection on the primary side, the stability of the output voltage is not affected, which is related to the overall mutual inductance. In addition, the high-frequency current in the uncoupled transmitters may result in extra power losses. Therefore, the uncoupled transmitters should be turned off. As for the control strategy for the segmented DWPT system [28], it is beyond the scope of this research paper.

## 4. Experiment Setup and Validation

### 4.1. Prototype Setup

**Figure 13:**
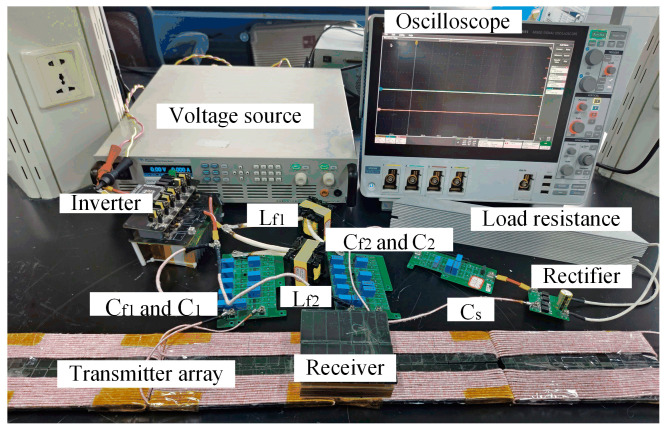
Prototype of the proposed DWPT system.

A 100 W prototype was constructed to verify the feasibility of the proposed DWPT system as shown in Figure 13, including a DC/AC inverter on the transmitting side with the MOSFET IMW65R048M1, resonant circuits, coupling coils, AC/DC rectifier with the diode 45R05SL, and load resistance. All the detailed parameters of the system are listed in Table 3. The input voltage of the system ranged from 60 V to 100 V, and the desired output voltage was 24 V. The resistance of the coils was tested by an LCR meter. The power analyzer HIOKI 3390 measures system efficiency and analyses power loss.

All the transmitters were identical and connected in parallel with a single full-bridge inverter which operated at a frequency of 140 kHz. The main coils were made from 350-strand AWG 38 Litz wire, and the ferrite material was PC40 from TDK.

### 4.2. Experimental Results

**Figure 14:**
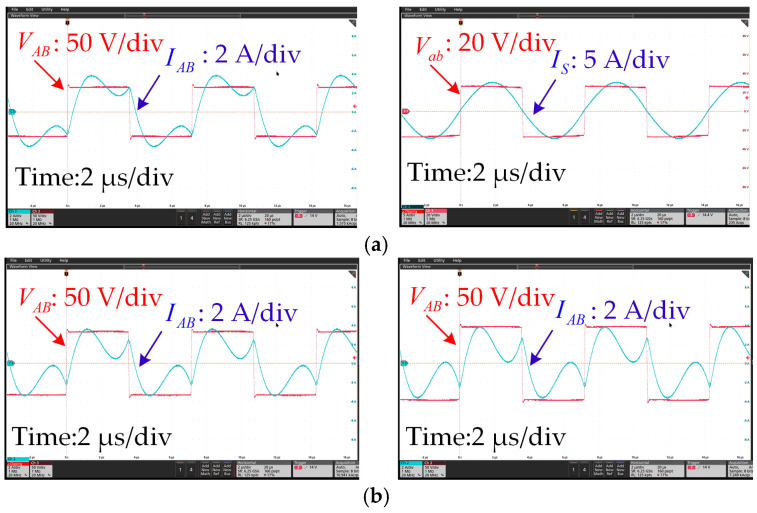
Experimental waveforms when *R*_L_ = 5 Ω. (**a**) Outputs of the inverter and rectifier when *h*_m_ = 20 mm. (**b**) Outputs of the inverter when *h*_m_ = 25 (left) and 30 mm (right).

The experimental voltage and current waveforms from when the receiver was fully aligned with the center of one transmitter, namely, the secondary transmitter *L*_P2_, with a 20 mm air gap, are shown in Figure 14. The input voltage was 65 V, and the load resistance was set to 5 Ω. Figure 14a shows the inverter output voltage and current waveforms. Figure 14b illustrates the inverter outputs when the air gap changed to 25 mm and 30 mm. The input voltage was set to 82 V and 100 V, respectively. Obviously, it can be seen that the output voltage *V*_AB_ and current *I*_AB_ were almost in phase, which means the inverter achieved a soft-switching condition. Therefore, the power loss of the inverter could be low. In addition, the rectifier input voltage *V*_ab_ and current *I*_s_ were in phase as well.

**Figure 15:**
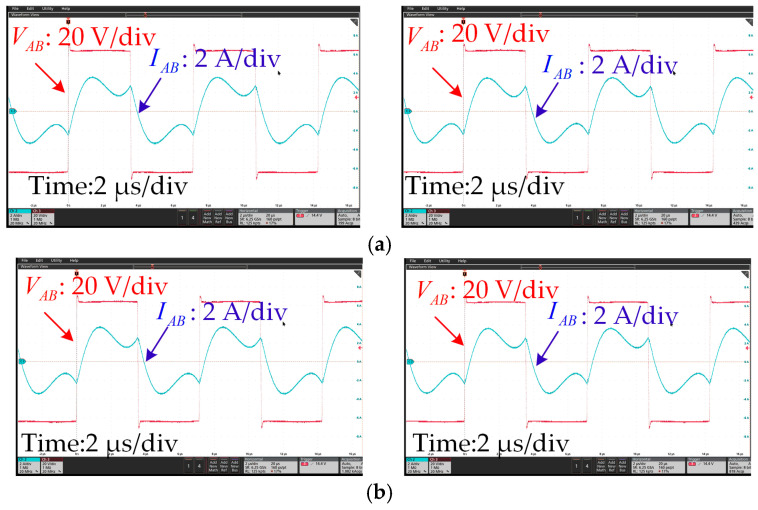
Experimental waveforms when *R*_L_ = 5 Ω. (**a**) Outputs of the inverter when *l*_m_ = 300 (left) and 350 mm (right) and (**b**) when *l*_m_ = 400 (left) and 500 mm (right).

Figure 15 shows the waveforms of the inverter when the receiver moved along the transmitter. It can be seen that when the *l*_m_ changed from 300 mm to 500 mm, the phase and amplitude of the inverter output voltage and current remained stable. The ZVS condition could be realized along the track.

**Figure 16:**
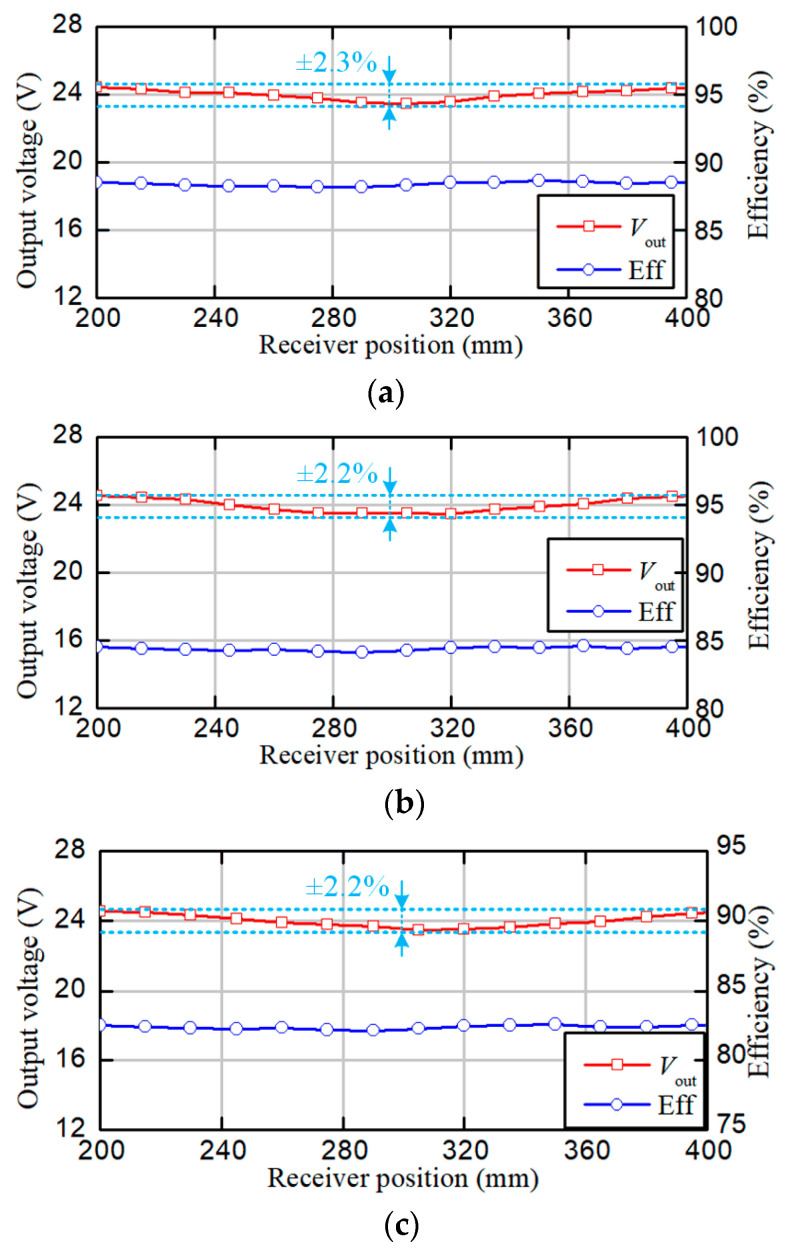
Curves of the output voltage and efficiency measured at different positions. (**a**) When *h*_m_ = 20 mm. (**b**) When *h*_m_ = 25 mm. (**c**) When *h*_m_ = 30 mm.

**Figure 17:**
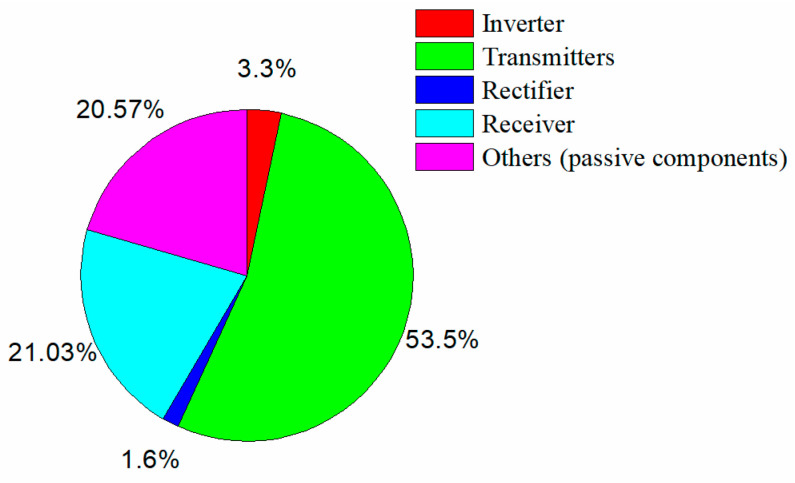
The power losses of the system when *h*_m_ = 20 mm.

The output voltage and efficiency from when the receiver moved along the transmitters are shown in Figure 16. Without the loss of generality, the receiver displacement *l*_m_ ranged from 200 mm to 400 mm. The air gap was set to 20 mm, 25 mm, and 30 mm, respectively. It is observed that the output voltage remained at 24 V with a variation of around ±2.3% over the operating region with different gap conditions. When the air gap was 20 mm, the output voltage fluctuated from 23.45V to 24.45 V. The overall efficiency ranged from 88% to 89%. The output voltage ranged from 23.5 V to 24.53 V when the air gap was 25 mm, and the efficiency was about 84.5%. When the air gap was 30 mm, the output voltage ranged from 23.5 V to 24.55 V, and the efficiency decreased to about 82.5%. This behavior is similar to the mutual inductance in Figure 8. The power loss in each component can be estimated, as shown in Figure 17. The parasitic resistances of the coils were measured as follows: $r_{Lp1}=0.33$ Ω; $r_{Lp2}=0.34$ Ω; $r_{Ls}=0.13$ Ω.

### 4.3. Comparison with Previous Works

**Figure 18:**
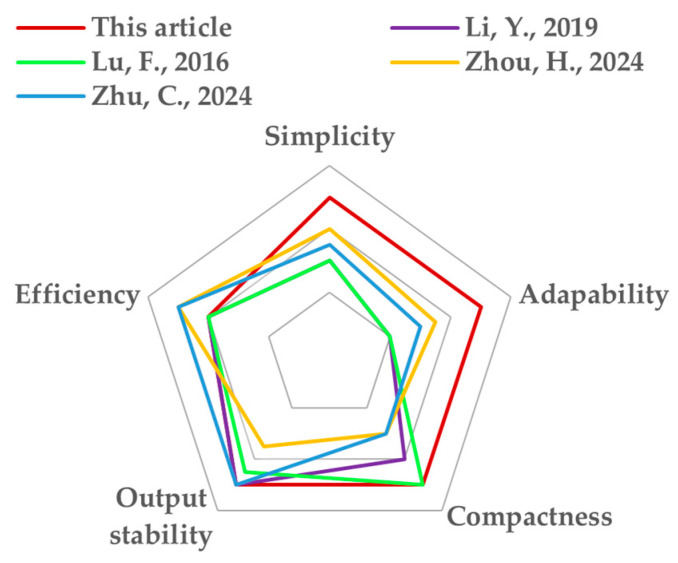
Comparison of various methods in [21,24,25,27].

The comparison between this paper and previous works is given in Table 4 and concluded in Figure 18. The simplicity index indicates the restrictions for the coil structure. For example, the proposed coil structure has few restrictions in the parameter design for achieving a stable mutual inductance. As for [25], the receiver size is strictly limited by the transmitter size and air gap. A stable mutual inductance cannot be guaranteed once the receiver size changes. Therefore, FEM simulation is often required for iteration optimization to find a specific set of parameters. The optimization process is complicated and time-consuming. The adaptability index means the ability of the system to adapt to different application scenarios or various charging requirements, such as for devices of different sizes or different air gaps. For this paper, the proposed transmitter structure can generate uniform magnetic fields regardless of the receiver size and gap distance, which is an outstanding advantage compared to other works. As for the other literature shown in Table 4, the receiver size is strictly limited by the transmitter size and air gap. A stable mutual inductance cannot be guaranteed once the air gap changes. The index compactness implies the arrangement of the coils and ferrites. The proposed transmitters are arranged closely for a larger coupling coefficient. And, the structure can be considered as normal Q coils. Correspondingly, in [27], more ferrites are required for I-shaped coils placed far apart. In [21], the transmitters are also placed far away from each other, which decreases the potential power density. The last two indexes are output stability and efficiency. The output fluctuation of the proposed system is ±2.3%, with an 88.5% efficiency. Obviously, the proposed structure has excellent performance in design freedom and adaptability compared to others. And, the output stability remains a high level, with a relatively high efficiency.

## 5. Conclusions

This paper has proposed a DWPT system to reduce power pulsations in the dynamic power transfer process for linear transport systems, which is suitable for magnetically levitated movers with a limited size. A new coil structure with terminal parts reversely bent has been proposed to construct the transmitter array. The design procedure of the coil is quite simple, without any iterative optimization by simulation which is time consuming. Further, the proposed coil structure maintains a stable mutual inductance with a ripple of no more than ±2.3% for various transfer distances. These performance characteristics were confirmed by a simulation and experiments. Thus, the proposed system can be conveniently assembled to create an open or closed travel path for power transfer. And, the transfer distance can be adjusted according to various working requirements. A 100 W prototype was constructed for verification. The efficiency from the dc source to the dc load reached 89%.

The proposed coil structure can be applied to various fields, such as electric vehicles, mining transport vehicles, and monorail cranes.

## Figures and Tables

**Table 1:** Parameters describing coil structure.

Symbol	Explanation	Symbol	Explanation
*l*_p_	Transmitter length	*l*_s_	Receiver length
*w*_p_	Transmitter width	*w*_s_	Receiver width
*h*_c_	Coil thickness	*h*_f_	Ferrite thickness
*h*_m_	Air gap distance	*l*_m_	Receiver position

**Table 2:** Dimensions of transmitters and receiver.

Parameter	Value	Parameter	Value
*l*_p_	200 mm	*l*_s_	80 mm
*w*_p_	100 mm	*w*_s_	80 mm
*h*_c_	2.5 mm	*h*_f_	5 mm
*h*_m_	20–30 mm		

**Table 3:** System specification and parameter values.

Parameter	Value	Parameter	Value
*V*_dc_	60–100 V	*V*_out_	24 V
*f*_s_	140 kHz	*R*_L_	5 Ω
*L*_f1_	20 μH	*C*_f1_	64.6 nF
*L*_f2_	20 μH	*C*_f2_	64.6 nF
*L*_p1_	59.84 μH	*C*_1_	36.8 nF
*L*_p2_	60.04 μH	*C*_2_	36.8 nF
*k*_p1p2_	−0.08	*L*_S_	36 μH
*k*_total_	0.1–0.18	*C*_s_	36 nF

**Table 4:** Comparisons of different methods.

References	Coupler Structure	Air Gap	Restrictions for Coupler Design	Output Stability	Efficiency
This article	Tx: reversely bent Q coil; Rx: Q coil	20–30 mm	no specific restrictions	24 V ± 2.3%	88.5%
[21]	Tx: Q coils; Rx: triple Q coils	50 mm	matched Tx and Rx parameter design	1000 W ± 3.49%	93.07%
[24]	Tx: Q coils; Rx: Q coil	150 mm	matched Tx and Rx parameter design	1400 W ± 2.9%	89.78%
[25]	Tx: DD + Q coils; Rx: DDQ coil	100 mm	matched Tx and Rx parameter design	96 V ± 2%	90.374%
[27]	Tx: I-shaped coils; Rx: Q coil	25 mm	matched Tx and Rx parameter design	1100 W ± 1.18%	87.98%

## Data Availability

The data can be accessed from this manuscript.
